# Physical, mental and healthcare issues of children on the street of Ibadan, Nigeria

**DOI:** 10.4102/phcfm.v15i1.3819

**Published:** 2023-05-26

**Authors:** Abimbola M. Obimakinde, Moosa Shabir

**Affiliations:** 1 Family Medicine Unit, Department of Community Medicine, Faculty of Clinical Sciences and Public Health, University of Ibadan, Ibadan, Nigeria; 2 Department of Family Medicine, University College Hospital, Ibadan, Nigeria; 3 Department of Family Medicine and Primary Care, School of Clinical Medicine, Faculty of Health Sciences, University of the Witwatersrand, Johannesburg, South Africa; 4 Department of Family Medicine and Primary Care, Faculty of Health Sciences, University of the Witwatersrand, Johannesburg, South Africa

**Keywords:** family, children-on-the-street, unhygienic, concoction, hunger, sexual, hawk, injury

## Abstract

**Background:**

Most street children studied in lower- and middle-income African countries are without family links. However, the majority of street children are children on the street, living with families during the night and spending their day-time on the streets. The health of this majority group is poorly captured in the literature despite the growing epidemic of child streetism.

**Aim:**

To explore the health of children on the street of Ibadan using multiple qualitative studies.

**Setting:**

A street in each of the five urban local government areas of Ibadan Oyo State, Nigeria.

**Methods:**

Participants comprising of children on the street, parental figures, street shop owners and child-welfare officers were purposively selected and interviewed. Interviews were audio recorded, transcribed, and thematically analyzed.

**Results:**

Using triangulated data from 53 interviews, the study found that the children on the streets of Ibadan experienced many health challenges. Outstanding are poor carbohydrate-based diet, open defaecation with consequent infections, physical injuries and few deaths from road traffic accidents. Sexual, verbal and substance abuse were common although few children acquired resilience to adversity. The children had poor health-seeking behaviour and resorted to patent medicine dealers or tradomedical practitioners on the streets.

**Conclusion:**

This study bridged some gaps in the literature regarding the health of children on the streets in Nigeria. The straddling of children between the family and street has cumulative health consequences as depicted in this study.

**Contribution:**

This research can inform family-level intervention and primary health care plans to forestall the health challenges of children on the streets.

## Introduction

Children on the street have existing links to their families and constitute 80% – 90% of street children, in urban regions of low- and middle-income countries (LMICs), like Nigeria.^1,2,3^ These children return home daily to their families regardless of their street activities, unlike other minority groups of street children including children of street families, children of the streets and children who absconded from institutional care.^1,2,3^

In 2008, the United Nations Children’s Fund (UNICEF) reported a global estimate of 218 million cases of child labour, including 150 million estimates of street children in LMICs, particularly in Africa.^4,5^ The 2007 Nigeria Population Reference Bureau (PRB) estimated 15 million street children in the country with the majority as children on the street.^5^ A 2021 publication reported 5026 child labourers from a 7-day headcount exercise of children on the streets of Ibadan, Nigeria.^5^ The Volunteer Work Africa (VWA), in 2022, estimated 100 000 street children in Lagos, Nigeria, the majority of whom were on the street for reasons of parental poverty, separation or unemployment.^6^ Owobu and colleagues, in 2022, reported that 9 out of 10 000 adolescents in Edo State, Nigeria were street children with existing family ties.^6^ These estimates are insights into the prevailing phenomenon of child streetism.

Children’s health problems on the street can be attributed to family food insecurity, street environment, interactions and healthcare access. Extensive research on the health of street children pertains to children of the street with severed family links, but the health of the children on the street is not outstanding.^1,2,3,7,8,9^ Health problems of street children include malnutrition, infectious diseases, substance abuse, sexual abuse, injuries, behavioural disorders and a lack of access to health care.^1,2,3,7,8,9^ A meta-analysis of African literature revealed the aforementioned morbidities among street children and recognised that most studies were centred on children of the street.^2^ Another systematic review revealed that the studies concentrated on minority groups of street children lacking specifics for children on the street.^10^ In a health study of street children conducted in Ibadan, Nigeria, although 90% were acknowledged as children on the street, they mostly captured children of the street.^11^ The study then recommended dedicated research to explore the health of children on the street who are the major category of street children.^11^ Considering that children on the street are the prominent category, the lack of data on their health constitutes a disparity in health research on street children.^1,2,3,7,8,9,12^ The 2015 report on ‘Children Left Behind in Nigeria’ and similar reports recommended targeted research to ameliorate this disparity.^13,14,15^ This study explored the health of children on the street of Ibadan, sourcing in-depth information and providing insight on specific health problems.

### Theoretical framework

The Bronfenbrenner bio-ecological system theory was used for the conceptualisation of this research. This theory examines the interaction between factors in the immediate family and society that can affect the child’s development.^16,17,18^ The theory addresses the microsystem, mesosystem, exosystem and macrosystems of a child.^16,17,18^ The family is the typical microsystem with the unusual ecological crossing into the streets that are an atypical microsystem, where children mingle with other elements outside the family.^16,17,18^ In the exosystem, the child welfare officers are involved with the support and care of these children on the street, and street shop owners witness their livelihood. In describing the health of children on the street, there is a need to evaluate their immediate family and understand the interactions on the street that affect their health and take the perspective of persons in the ecosystems.

## Research methods and design

### Design and sampling

This descriptive cross-sectional study evaluated the everyday conscious understanding of the health experiences of children on the street while setting aside preconceived opinions.^19^

This research utilised four qualitative methods including in-depth interviews (IDI) with children on the street, paired IDI with a dyad of a parental figure and a child on the street and IDI with Oyo State’s child welfare officers and street shops or business owners. Participants were sampled purposively using the snowball technique from five streets in Ibadan, capital of Oyo State Southwest Nigeria, selecting a street from each of the five urban Local Government Areas (LGAs): North (N), Northwest (NW), Southwest (SW), Northeast (NE) and Ido. The selection of a street in each LGA was guided by information derived from the Oyo State directorate for child welfare services, which provided information on the streets with a known active population of children on the street. Sampling across the five urban LGAs of Ibadan allowed for a detailed description of the phenomenon in Ibadan.

### Participants and recruitments

The selection of children on the street was initially purposive as guided by the street shop owners who were familiar with the parental figure and thereafter by snowball technique on each of the streets. The sampling matrix as shown in Table 1 explains the participants’ characteristics and selection across each of the five urban LGAs in Ibadan.

**TABLE 1:** Sample matrix for recruited participants.

Number	Category of participants	Characteristics	Minimum selected in each of the five LGAs	Total in each category
1	Child on the street	Aged 13 years–17 years 11 months	2	11
2	Parent–child pair	One parental figure and a child on the street aged 13 years–17 years 11 months	2 Pairs	10 Pairs
3	Street shop owner	Street shop or business owner on select street Being a past child on the street was an optional desirable criterion	2	10
4	Welfare officer	Oyo State-appointed child welfare officer working in each LGA and the central directorate in Ibadan	2 per LGA and 2 from the Oyo state secretariat	12

	**Total**			**53**

LGAs, local government areas.

### Data collection procedure and measures

#### Ethical approvals were obtained

Data were collected between June and September 2021 and universal precautions (including the use of facemasks and hand sanitisers) against coronavirus disease 2019 (COVID-19) were ensured. The research document was written in English language and the Yoruba (local) translation. A back translation of the Yoruba version to English was done to ascertain the literal validity of the original document. Either of the two versions was used based on the language preference of the participants. Ibadan city has a mix of indigenous mostly Yoruba-speaking people and educated people who can converse in English. Written consent and assent were obtained from each participant. The interviews were moderated using a pre-developed interview guide and audio-recorded using a digital recorder. The interview guide was self-developed to explore the pertinent health issues of children on the street referenced in literature and to fill the gaps identified. The interview guide adopted probe questions that had been used by other studies to evaluate the documented health concerns of children on the streets, emphasising on their health status and challenges.^2,4,11^ The guide contained questions exploring general health problems, problems regarding food and water consumption, toilet habits, mental health-related problems, health hazards on the streets, healthcare-seeking behaviours and options of children on the street.

### Data management

Direct and complete transcripts of the audio-recorded interviews were typed out in Microsoft Word. The qualitative data from the 53 interviews were analysed using procedures of framework analysis, including five levels of systematic data processing: familiarisation, identification of thematic framework, indexing, charting and mapping. The data from the four qualitative studies were triangulated^19,20^ by converging information from all participants. Coding was done using the Atlas Ti version 8.4, with the generation of specific codes for a robust appreciation of the health issues of children on the streets of Ibadan.

### Ethical considerations

Ethical clearance to conduct this study was obtained from the University of Witwatersrand and the Oyo State Ethics Review Committee (No. M210424 and AD 13/479/4118A).

## Results

The analysis was organised into three themes: (1) physical health challenges, (2) mental health issues and (3) healthcare challenges. The physical health challenges included: (1) poor nutrition, (2) poor toilet habits, (3) exposure to infections, (4) sexual health problems, injuries with body aches and allergies. The mental health subthemes included: (1) depressive feelings, (2) misbehaviours, (3) verbal abuse, (4) substance abuse and (5) psychological strength. The healthcare subthemes included: (1) poor health-seeking behaviours and (2) unconventional healthcare.

These themes emerged through framework analysis of the triangulated data obtained from the children and significant others within their ecosystem. The health conditions were consequent of cumulative factors in the immediate family and the street microsystems. Poor nutrition, depressive feelings, verbal abuse, poor health-seeking behaviours and unconventional healthcare stemmed from factors within the family and are worsened by the children’s presence within the atypical street microsystem where they spend most of their daytime. Poor toilet habits, exposure to infections, sexual health problems, injuries with body aches, allergies, misbehaviours, substance abuse and psychological strength were consequences of the unusual ecological crossing into the streets where children mingle with other elements outside the family. Table 2 describes the respondent labelled in the bracket for each quote.

**TABLE 2:** Codes for the respondent groups.

Number	Abbreviation	Description
1	C	Child on street
2	F	Female
3	M	Male
4	PC	Paired child
5	PP	Paired parent
6	SO	Shop owner
7	*SO	A shop owner who was previously a child on the street
8	WO	Welfare officer

### Physical health challenges

The physical health challenges are physical conditions affecting the children on the street due to street elements, some of which are underscored by factors within the family. Six aforementioned subthemes explained these physical health challenges including poor nutrition, poor toilet habits, exposure to infections, sexual health problems, allergies and injuries.

#### Poor nutrition

Many of the children who were interviewed reported that they were unable to access food in their household and went hungry until they made money from street hawking or their parents received handouts from neighbours. Child welfare officers shared instances:

‘The mother leaves early in the morning without providing food for the children and returns home at 11pm, expecting the children to look up to their neighbours for food. The children just find their way to the street.’ (WO5,46, F)

A few children shared their stories:

‘When we wake up in the morning we do house chores, then whatever little money our parent has, they give us to buy something to eat first, maybe later if they see someone to give them money we will eat properly.’ (PC7,17, M)

‘I can only buy food to eat after making a sale.’ (PC3,13, M)

‘I do buy food to take home for us to eat because my parents don’t have much money to get food for the family.’ (C10, 17, M)

Shop owners said that the meals available and affordable to these children were mostly carbohydrates and junk food, which the children prefer because it is filling. The children and parents gave examples of regular meals consumed by the children:

‘My mother gives us only bread for breakfast daily, my older brothers work in a bakery so they bring bread home. When I carry loads on the street, I make like two hundred naira and buy beans to eat the bread and by evening I make more money to cook the food I like.’ (PC7,17, M)

‘He buys and eats rice or bread when he is hungry while hawking on the street, he eats bread in the morning before leaving.’ (PP3,35, Mother)

‘I buy and eat rice, biscuits and doughnuts every day.’ (C2,14, M)

Street shop owners and welfare officers mentioned that the children drank from various sources of water, including hygienic ones like pre-packed treated water. It was, however, mentioned that the children drank untreated sachet water and unhygienic ones like stagnated well or rain water when on the street. A few of the children who hawk *pure water*, that is pre-packed sachet water, reported that they drank from their stock when thirsty. Participants opined that the children buy sachet water, because it is cheap or take water from government boreholes or well water on the street:

‘They drink whatever type of water that is available, clean or not, it doesn’t matter as long as it quenches their thirst.’ (WO3,41, F)

#### Poor toilet habits

Shop owners and welfare officers were disturbed about the poor toilet habits of the children and discussed it at length. They mentioned that the children urinated indiscriminately and indulged in open defaecation. Welfare officers shared instances of indiscriminate defaecation including defaecation into polythene bags, tied and left on the streets:

‘We don’t have a toilet on this street, the children go inside the bush where the breeze blows freely, and they use the leaves in the bush to clean up.’ (SO8,28, M)

A few of the children and parents mentioned that the children go home to use the toilet facility or defaecate in nearby bushes, rivers and streams:

‘There is no toilet on this street. He would hold it till he gets home but if he is pressed, he goes to defecate in the bush at the back of that factory.’ (PP3,35, Mother)

‘He said he went to a stream to defecate, and his other sandal got carried away by the stream.’ (PP8,38, Mother)

Some adults strongly opined that indiscriminate defaecation by the children is because of the inadequate number and cost of use of public toilet facilities on the streets of Ibadan:

‘It is an annoying fact that children on the street take to open defecation because the number of public toilets in the state is not enough.’ (WO12,51, M)

‘There is a toilet somewhere in the market. It is 50 naira to defecate and 20 naira to urinate and he can’t pay that every time.’ (P10,60, Grandmother)

#### Exposure to infections

Shop owners and welfare officers reported exposure of the children to faecal-oral diseases and other infections:

‘They commonly have rashes on their body and scalp. Malaria is common since they always walk and get bitten by mosquitoes from those stagnant gutters and during this COVID-19 [*coronavirus disease 2019*] era, they can easily catch it.’ (WO8,43, M)

A few respondents expressed concerns about the unhygienically prepared and vended food on the streets and the risk of gastrointestinal infections:

‘Presently a constant variable is the unhygienic condition these children found themselves in. There is poor environmental sanitation and a lack of proper waste control on most streets which readily contaminates the food and water they consume, and they get sick.’ (WO12,51, M)

Parents and children expressed their fear of children contracting infections and gave examples of instances of illness:

‘We have helped people carry loads and used our hands to pick their oranges from the gutters, we carried loads dirtied in mud and disposed of people’s waste on our heads, I think that gave me diarrhoea one time.’ (PC7,17, M)

‘It is God that protects against food contamination and diarrhoea, we eat on the street but as a mother, I know it is not proper and responsible of me to have my child hawking and eating from street canteens which are usually dirty, but it is because of our situation.’ (PC,35, Mother)

A few children reported that in their opinion they had contracted genitourinary infections like gonorrhoea through indiscriminate urination in dirty places and child welfare officers recounted similar cases:

‘I can “toilet” (defecate or urinate) anywhere, to the extent that I have “toilet disease” many times.’ (C8,17, F)

#### Sexual health problems

Shop owners and child welfare officers passionately shared stories of sexual exploitation, abuse and assault of both boys and girls with consequent teenage pregnancies. Shop owners reported that physically mature girls get sexual advances and harassment from men and reported sexual exploitation between girls and boys on the street:

‘It happened to me when I hawked as a child, “area boys” see me and say: “girl how far?” Back then, I took alternative street routes to avoid the area boys.’ (*SO7,41, F)

‘The girl was at a tender age of eight years. I visited the mother and intervened for her to be taken off the street because she has been sexually abused repeatedly.’ (WO3,41, F)

Welfare officers narrated cases of young girls who hawk and have been repeatedly sexually abused by men and shared stories of unwanted teenage pregnancies:

‘Girls’ population is not much among the children, but unwanted teenage pregnancies are common nowadays. Sometimes ago we went out for raiding children, unfortunately, some girls had positive pregnancies during the routine test we did.’ (WO12,51, M)

‘A case I handled before, was that of a girl who was repeatedly sexually abused, and nobody took responsibility when it resulted in pregnancy.’ (WO5,46, F)

Some mothers said that they avoid sending their mature daughters to the street to avoid sexual exploitation. A few shop owners and welfare officers opined that unwanted teenage pregnancies and births contributed to the increasing population of children on the street:

‘Ibadan is the worst place; you see all those young girls hawking “pure” [*sachet*] water with the pregnancy. The boy that impregnated her will be doing “guy” around and still impregnate another girl.’ (SO8,28, M)

#### Aches and injuries

Most of the children complained of headaches that were worse on sunny days and pains affecting the neck and limbs. Some parents attested to these complaints and welfare officers and shop owners shared their awareness. Street shop owners who were previously children on the street narrated lingering body aches and pains dating back to childhood days of intense street hustling activities:

‘You know the kind of work I do, and I have been under the sun since morning, I usually have headaches or stomach aches, so that is how it is. Once in a while, I have body aches, leg pain, and shoulder pain.’ (PC7,17, M)

‘The load I am carrying give me a headache and neck pain … after making my hair newly then I still have to place the load on my head to hawk, I get headaches, worse under the sun.’ (C8,17, F)

‘My body aches generally, so my mother buys pain relief drugs for me almost every day.’ (PC4,17, M)

Street shop owners and children shared that they acquired injuries inflicted on each other from play or fighting among themselves or when physically assaulted by street thugs during forceful exploitation:

‘But the injury on his face was four days ago, he said his colleague threw a stone at his face.’ (PP8,38, Mother)

‘Those mature thugs beat them and collect their money, they make them suffer and wound them.’ (SO3,35, F)

Many children had scars and bruises on the face and limbs that they shared were the results of injuries sustained while playing or during other street activities, some of which were related to vehicle accidents. Child welfare officers gave accounts of children involved in road traffic accidents (RTAs) while hawking on the street; most had minor injuries and a few deaths were reported:

‘Moving car tyres stepped on him about a year ago and one of his big toes was injured and now deformed.’ (PP5, 30, Mother)

‘“Maruwa” (tricycle) hit me by the side of my body once and knocked me down, it did not break my bones although it weakened me.’ (PC7, 17, M)

‘A child died at Aleshinloye junction, he died as he was hawking and crossed the road in hurry and many of them had died like that.’ (WO9,56, F)

Welfare officers reported cases of children on the street who died from swimming when they went to defaecate in streams or decided to take a swim:

‘They leave their goods somewhere and go swimming in the streams around for fun and hope to catch fish and hawk it for money, unfortunately, some have drowned and died.’ (WO3,41, F)

‘There was a situation whereby some children went to the river to swim and drowned recently and two years ago at “Asejire” river.’ (WO12,51, M)

#### Allergies

A few children complained of eye irritation, chest discomfort and cough when on the streets. Some mothers acknowledged that these irritations occurred because their children were exposed to dust and fumes on the streets. Child welfare officers mentioned that allergic conditions were known health problems for these children:

‘I have red eyes, this one started last week. It gets worse when cars that emit a lot of heavy smoke pass by.’ (PC5,17, M)

‘Clouds of dust from passing vehicles affect my breathing at times.’ (C11,17, M)

### The mental health issues of children on the street

This is the second theme that entailed the mental health problems and strength found among the children on the street. The mental health theme has five subthemes, including depressive feelings, misbehaviours, verbal abuse, substance abuse and psychological strength.

#### Depressive feeling

A few children said that they occasionally felt ashamed, sad and hopeless when they thought of the situations that brought them to the street or saw other children being cared for by their parents passing by on foot or in cars. Child welfare officers share their concern for future depression in these children, especially those exploited and abused:

‘When I see my ex-secondary schoolmates in traffic while hawking, I don’t greet them because I feel shame.’ (C10,17, M)

‘I just don’t like hawking on the street like this. Shouting all about the street, while I am hawking so people can hear and patronize me, makes me unhappy.’ (PC4, boy,17)

#### Misbehaviours

Various misbehaviours of children were cited by shop owners and parents. A few of the children owned up to misbehaving towards each other, adults on the street and at home:

‘I tell my son “Why do you misbehave, why do you want to trouble my life?”’ (PP7,42, Mother)

‘Some of us do beat ourselves, we fight ourselves and throw away each other’s goods in the process.’ (PC1,16, M)

‘The only thing that bothers me and saddens my heart is that sometimes I misbehave towards my mother, and I tend to talk rudely to her.’ (C11,17, M)

Street shop owners and welfare officers said the children’s misbehaviours reflected their exposure to ill-mannered street thugs or touts:

‘Some of them learn how to fight on the street, they easily get angry, and they don’t have the habit of saying sorry when they offend someone.’ (SO7,41, F)

#### Verbal abuse

Some children reported verbal abuse from people on the street and described the unfair insults and derogatory remarks from people who patronised them. They reported being cursed and talked down to by customers, shop owners or passersby:

‘Customers do abuse me, insult me and they sometimes talk harshly when chasing me away from their cars and those things pain me.’ (PC6, 16, M)

‘A lot of people say to me that I do not have a mother or father just because I am hawking. They don’t know me, and they say that to me and it’s painful.’ (PC3,13, M)

A few children shared that their parents verbally abuse them at home and child welfare officers gave instances that they witnessed:

‘If I should carry my goods home and I didn’t make enough sales, my parents will be blaming me and shout at me when I get home.’ (C1,17, M)

‘When some mothers are talking, they say “the unfortunate child has gone hawking since morning and if the money is not complete, I won’t give him food”. You can imagine what they say directly to the child.’ (WO9,56, F)

#### Substance abuse

The children and their parental figures denied substance abuse among the selected children, but the shop owners and welfare officers reported that children are lured into substance abuse through their interaction with commercial drivers in garages and members of the National Union of Road Transport Workers (NURTW):

‘The smaller children would have been eyeing those smoking out of curiosity, so they eagerly taste. The older children are sent on errands by these street thugs to go and buy Indian hemp, “Oja” that’s their language and they eventually start smoking by association.’ (WO5,46, F)

‘I saw my mates drinking alcohol and I thank God I am not drinking alcohol.’ (C7,15, M)

It was reported that children smoke and drink all sorts of things that cause serious intoxication. Reportedly both boys and girls are lured into ingestion of ‘health’ herbs soaked with alcohol:

‘Children assist those selling alcoholic drinks on the street, particularly those who sell herbs soaked with alcohol called “Agbo – jekomo,” they are invited to taste it as a health remedy, and little by little they start taking alcohol habitually.’ (WO3,41, F)

Child welfare officers and shop owners opined that these children were at risk of substance addiction and related long-term mental health problems and they gave examples. Welfare officers reported the challenges of rehabilitation of the children once they got addicted; some skipped school and eventually left home:

‘We do see one child that had sold things in traffic on this street before, he has gone mad because he smokes Indian hemp, and he still roams the streets to date.’ (SO9,45, F)

#### Psychological strength

A few child welfare officers opined, that despite tales of woe, hazardous exposures and bad experiences, a minute proportion of children turned out to be mentally strong, vigilant and resilient:

‘Most of these children are psychologically strong. They are very determined, so they developed a kind of resistance to the way and situation they have found themselves in.’ (WO12,51, M)

‘I have “brain” no man can deceive me that he wants to give me five hundred, because I know that when I sell, I can make a gain of five hundred and I have seen results of girls that were deceived.’ (C8,17, F)

A few past and current children on the street shared that they learnt from the mistakes of their peers and were determined not to fall victim:

‘I had determination in my life to succeed because when I looked at my family and what brings me down in the family, I told myself anything bad I don’t want in my life and I won’t do it to myself. So, I distanced myself from bad friends and it helped me to focus on my life, now I am a degree holder.’ (*S10,34, M)

### Healthcare issues of children on the street

This third theme addressed the healthcare issues of children on the street. Two subthemes emerged including poor health-seeking behaviour and unconventional healthcare options available to the children on the street when they fall ill as a result of engagement with street activities.

#### Poor health-seeking behaviours

It was reported that the children had poor health-seeking behaviours. Shop owners and child welfare officers said that mothers downplayed these children’s illnesses and delayed getting treatment:

‘He doesn’t use drugs like that, except when he is very sick, he takes after me in that manner, when he has pains, he sleeps it off and wakes up alright.’ (PP6, 52, Mother)

‘The market women will ask the child “did you not know you were sick before coming out to the street?” and the child usually says, he knows, he just said he should manage to come and hawk.’ (SO7, 41, F)

Children living with guardians reported that the guardians disregarded their complaints of ill health, so they resorted to taking part of the money made on the streets to obtain drugs at the chemist without the knowledge of the guardian when the illness worsens. A few children said they found a place to nap or rest on the street when sick and thereafter continued their activities without getting any treatment:

‘When I have a headache, I pour cold water on my head, relax for 20–30 minutes it goes away and I stand up to continue my work.’ (C8,17,F)

#### Unconventional healthcare

Many respondents said that ill children on the street had limited access to conventional healthcare services and resorted to the available and affordable unorthodox options. A visit to the ‘chemist’ or ‘patent medicine seller’ is reportedly a popular option:

‘The child goes to the chemist and explains the illness. The chemist won’t ask if he or she has eaten or not, they take money from the child and count different kinds of drugs and hand it over. Even when the mother is informed all she does is buy an over-the-counter mixture of pills from a chemist and give the child.’ (WO6,54, F)

‘I will get drugs by myself at the chemist, I tell the chemist how I feel and they give me whatever drugs I need. Sometimes, I could have remnant drugs at home from the chemist and I use just that.’ (C9,17,M)

Street shop owners reported that even when children go home or tell their mothers of their illness, the treatment option includes a visit to the chemist or purchase of pills from roadside drug peddlers. Child welfare officers opined that because the children are minors, they are unable to approach conventional health care providers and opt for herbal concoctions, which are readily accessible and affordable. Welfare officers said that the mothers preferably look for tradomedical practitioners or procure herbal concoctions for the child:

‘I use herbal drugs, I buy from herb sellers that hawk around the street, they already know how they sell it to me.’ (C10,17, M)

‘They [*children*] tell the herb seller their complaints and the seller will mix different concoctions and give them to drink on the spot.No quantity or no dosage specifications.’ (WO9,56, F)

‘We were given the herbal concoctions from the tradomedical place and directed that he should take it with hot water regularly so that it will reduce it.’ (PP9,40, Mother)

## Discussions

This research explored the health issues of children on the street of Ibadan via interviews with the children and adults in their ecosystem. The results revealed expected health challenges and realities not yet published.

### Physical health challenges of children on the street

Food security is defined based on the availability of adequate food, access to food and appropriate food consumption.^21,22^ The children on the street experienced occasional household hunger; they had poor feeding patterns, and carbohydrates were the available staple foods for them in their homes. Specifically, it was deduced that most of the children barely had breakfast daily or ate plain bread, and they bought cheap food that contained mainly carbohydrates when on the street. This research revealed that many of these children lived in a household with inadequate quality and quantity of food that can support their nutritional requirements for proper growth. Food insecurity in Nigeria is associated with the poor earning power of the head of the family,^22^ which is relatable in this study. This is because the money generated by the children is needed for the children’s breakfast or to support the family’s main meal, which is usually dinner. This study revealed that children on the street are from food-insecure families, clearing the impression of a better nutritional status among this category of street children.^10,23,24^

Although in this study many of the children drank pre-packed ‘sachet’ water that is affordable, it is common knowledge in Nigeria and other neighbouring countries that not all ‘sachet’ water is hygienically produced.^11,25^ The tendency for these children on the street of Ibadan to drink ‘sachet’ and other unclean water with the attendant contamination of food and water due to the unhygienic nature of the street can cause diarrhoeal diseases as found in this research and, published previously, for children living on the street.^26,27^

The unhygienic and indiscriminate sanitary habit of children while on the street was extensively reported, while open defaecation featured prominently as similarly published for street-dwelling children in other sub-Saharan African LMICs.^11,28,29^ Interestingly, this study found that few children go back home to use the toilet facilities when convenient. Likewise, the inadequate number and fees charged for public toilets in Ibadan have not been extensively published as a reason for the indiscriminate toilet habit of children on the street. The defaecation of children into streams or rivers with the risk of drowning and death was an unexpected finding.

The attendant risk of communicable febrile illnesses like malaria and COVID-19, food and water-related gastrointestinal disease and skin rashes were not exceptional findings in this study. In the background of poor nutrition and the unhygienic nature of the streets in LMICs, children on the street are prone to food, water, insect and air-borne diseases.^10,26^ The lack of personal protective devices during a pandemic as observed in this study is another factor that can predispose children on the street to communicable diseases. Street children have been described as invisible and excluded from the health care plan.^12,30,31^ This was apparent during the data collection for this study, as despite the COVID-19 pandemic, there were no targeted plans or actions from the health ministry for the protection of children on the street. These children were unperturbed about masking up until convinced by the research team. Related to air-borne disease exposure is the risk of environmental health hazards, like the dust raised by passing vehicles and smoke from exhausts that resulted in irritative allergic symptoms for children in this study. The report on eye and lung allergic reactions among children on the street is insightful and should be a focus for future health research. Although studies had reported inhalation injury among homeless street children and respiratory infection as morbidities among children of the street, there is no mention of allergic respiratory disease.^32,33^

Many of the children in this study complained of recurrent injuries, aches and pains, which were attributed to musculoskeletal strains caused by activities on the street, as expected.^10,26^ Common causes of physical injuries for children including fights with peers, trauma sustained due to roughness of the street and motor vehicular accidents were reported in this study.^29,33,34^ These causes of injuries were reported for the children in this research. Nevertheless, it was reported that boys get injuries inflicted due to physical assault while being financially exploited by unscrupulous motor-garage men. Financial exploitation particularly of the boys on the street had been reported by other African studies.^35,36^ However, the fact that the process of financial exploitation by street thugs inflicts physical injuries on the children on the street was an interesting finding in the research. Road traffic injuries (RTI) were featured in this study as expected and a few deaths from RTI were unfortunate incidents. The safety of these children should never be undermined, and understandably interviewed mothers were very concerned about the risk of RTI but seemed incapacitated by their needs for the income generated by these children.

In this study, children hawking goods on their heads complained of neck pain, and former children on the street complained of chronic low back pain, dating to childhood days of hauling loads on the street. These musculoskeletal complaints suggest that neck and back arthropathy should be considered as part of the health challenges of children on the street. Body aches and pains when associated with headaches and malaise could be symptoms of malaria considering their exposure to incessant mosquito bites from stagnant water on the street.^37^ A few of the children who reported that they needed to rest and nap somewhere on the street could have had malaria-related aches and pains.

This study found that children on the street of Ibadan were sexually harassed, exploited and abused. A study published that new and younger children were prone to sexual abuse by adults or older boys and girls on the street, contrary to reports from other studies, as opposed to other literature on street children.^36^ The sexual exploitation reported in this study was recurrent regardless of gender, age of the children or time of arrival on the street, as opposed to other literature on street children.^37,38,39^ In this study, sexual exploitation with consequent teenage pregnancies and refusal to identify or ascertain the perpetrators were reported. Likewise, denial of paternity from men or boys that impregnated the girls was a common event on the streets of Ibadan. Accordingly, Fawole and colleagues published that society may show sympathy for sexually abused girls, but there is an attendant risk of stigma, fear or shame that deter them from reporting the sexual abuse.^40^ The study further revealed that street girls who report the abuses are prone to physical assaults by perpetrators and loss of their goods, among other things, and these might be the reasons why girls were reluctant to report sexual abuse to date. Out of concern about sexual exploitation and its consequences, a few mothers in this study preferred not to send their mature daughters to the street, whereas some mothers were unbothered. As was expected, there were few reports of sexually transmitted infections (STIs) among children in this study.^26,37,40^ However, the report of children in this study, who had contracted sexually transmitted and urinary tract infections from indiscriminate and unhygienic toilet habits on the street needs future probing.

### The mental health of the children

The children in this study reported depressive feelings in the form of shame, sadness and hopelessness associated with their situation. These feelings are related to the reasons for their presence and activities on the street. Published literature on the mental health of street children has reported mood disorders but specifics for children on the street are not obvious as highlighted by this study.^36,41,42^ Behavioural problems are documented mental health challenges of other minor categories of street children and there are gaps in the literature regarding behavioural problems among children on the street.^3,43^ Therefore, it was enlightening to find children in this study who realised their misbehaviours towards their parents and worked on positive change. Additionally, street shopkeepers in this study had understudied the trajectories of behavioural problems of these children. The shop-keepers described a trajectory that typically began with discourtesy and aggravated to unruliness and aggression.

Verbal abuse from parents at home was a discovery in this study, which occurred when the children were unable to meet the parents’ expected sales target on the street. The children had to endure constant cursing and derogatory remarks from people who patronise them and other people who would not patronise but swear at them at the slightest chance. Although studies on the mental health of street children had highlighted general emotional problems mostly in children of the street, verbal emotional abuse was not prominent.^24,33,44,45^ The complaint of verbal emotional abuse must not be trivialised, because if it coexists with feelings of shame, sadness and hopelessness it may become a risk for depression and substance abuse among children.

The use and abuse of alcohol and smoking of marijuana (*Oja*) were reported among children in this study. Although all interviewed children denied substance abuse, some gave examples of their peers involved in this act. The presence of children on the street and exposure to unscrupulous adults, particularly commercial motor-park touts (*Agbero*) as reported in this study, was concerning. The association of these children with the *Agberos* constituted a risk for their substance abuse particularly marijuana.^11,35,42,46^ A study pointed out the lower prevalence of unprescribed medication and substance abuse when children stay and are cared for at home and are not exposed to street life.^47^ Smoking marijuana, alcohol use and abuse are known mental health problems of street children in general.^11,35,42,46^ However, this research detailed the manner of indoctrination of children on the street bridging research gaps on substance use among street children.

It was a pleasant surprise that few participants in this study opined that positive mental health attributes can be developed by children. These attributes included resilience and determination to succeed, as previously documented for children of the street.^48,49,50^ Although this study did not specifically enquire about the factors that support the positive mental health attributes observed in the children, it was inferred that they learnt from other people’s mistakes. Studies have associated resilience in homeless street children with support from non-governmental organisations (NGOs), religious beliefs, meaningful and reciprocal friendships and a sense of humour.^42,49^ However, there are many adverse health issues for the child on the street, which outweigh these perceived mental health benefits, as listed in this study and corroborated.^50^ This perspective of positive mental attributes gained by the children does not excuse the fact that the street is an unhealthy microsystem for the physical, cognitive and psychosocial development of a child.^36,51^ A child raised within a family can be linked to other microsystems like school and other ideal social networks where these positive mental health attributes can be developed and nurtured.

### Healthcare challenges

In this study, the children delayed seeking healthcare because they or their mothers trivialised illness symptoms, and at the extreme illness, complaints were ignored by the guardians until it becomes critical. It was reported that one of the reasons for the delay in healthcare seeking is because the mothers lacked knowledge of orthodox healthcare. Eventually, when they did seek healthcare, they preferred unorthodox healthcare due to its accessibility, affordability and alignment with parents’ beliefs. A 2008 Indian study revealed similarly that parents and associates of street children, the severity of the illness and the family’s financial constraints were obstacles to prompt health-seeking of working street children.^52^ Although that study included all working street children and did not delineate the obstacles for children on the street, as found in this study. Another study in Durban reported poor health-seeking behaviours among homeless street children, which were associated with non-existing links to a family, which could render them the needed support to seek prompt healthcare.^50^ This study has shown that children on the streets have poor health-seeking behaviour related to socioeconomic factors and the health perception of the family.

Preference for unorthodox and unconventional healthcare is the norm for the children in this study. The patronage of a chemist, street drug vendors and the use of herbal concoctions by children on the street of Ibadan is not a new finding.^37,53^ However, what is worrisome is the normalisation of consumption of herbal concoctions and alcohol-based ‘health herbs’ among these children, which is being vended by women on the street. A study had queried the apparent ‘innocent’ vending of harmful substances to children on the street by grown women who should be better informed.^35^ Therefore, the national monitoring agency for drug administration and child welfare officers needs to regulate sales of health herbs especially alcohol-based ones to street children and increase awareness of the health hazards. Unmonitored patronage of untrained patent drug-store owners on the street because of proximity and affordability exposes the children to consuming mixtures of over-the-counter pills with its attendant acute and chronic systemic health problems.

### Strength and limitations

This study, to the best of the authors’ knowledge, revealed the realities of health issues of children on the street of Ibadan, Nigeria that have not been explicitly written in literature. In addition, various opinions were sought from divergent stakeholders. However, a limitation is the fact that most of the mentioned infectious diseases could not be substantiated by specific objective assessments and measures.

### Implication for further research

Further research on the health issues of children on the street is recommended, incorporating validated quantitative measures that can further substantiate the health problems of these children within the provision of research ethics of vulnerable children.

## Conclusion

This study revealed the health issues of children on the street of Ibadan Nigeria using a qualitative approach by sourcing information from the children and significant informants in their ecosystem. The result showed that the health issues identified are more related to their presence on the streets, which is an atypical microsystem for these children. Therefore, family-level interventions are necessary to deter the family-oriented health challenges and control the street-related health problems and adverse exposures. For instance, the poor nutrition, mortality related to poor toilet habits and RTI, the luring of children to marijuana and alcohol use by street touts and unrestricted sales of alcohol-infused herbal remedies to children should be curbed.

## References

[1] Alem HW, Laha A. Livelihood of street children and the role of social intervention: Insights from literature using meta-analysis. Child Dev Res. 2016;2016:3582101. 10.1155/2016/3582101

[2] Cumber SN, Tsoka-Gwegweni J. The health profile of street children in africa: A literature review. J Public Health Africa. 2015;6(2):85–90. 10.4081/jphia.2015.566 PMC5349275 28299148

[3] Mounir G, Attia M, Tayel K. Street children in Alexandria: Profile and psychological disorders. J High Inst Public Health. 2007;37(1):56–77. 10.21608/jhiph.2007.22014

[4] Abdullah M, Basharat Z, Lodhi O, Wazir M, Khan H, Sattar N. A qualitative exploration of Pakistan’s street children, as a consequence of the poverty-disease cycle. Infect Dis Poverty. 2014;3(1):1–11. 10.1186/2049-9957-3-11 24661542 PMC4022352

[5] Taiwo AO, Badiora AI, Adebara TM. Public spaces and concentrations of child labourers in Ibadan Municipality, Nigeria. J Asian Afr Stud. 2021;56(7):1708–1723. 10.1177/0021909620988747

[6] Yusuf M, Tsagem S. Empowering street children in Nigeria: Non-formal education and counselling potentials population. SER. 2022;21(1):1–12.

[7] Abubakar-Abdullateef A, Adedokun B, Omigbodun O. A comparative study of the prevalence and correlates of psychiatric disorders in Almajiris and public primary school pupils in Zaria, Northwest Nigeria. Child Adolesc Psychiatry Ment Health. 2017;11(1):1–12. 10.1186/s13034-017-0166-3 28596800 PMC5463351

[8] Oppong Asante K. Street children and adolescents in Ghana: A qualitative study of trajectory and behavioural experiences of homelessness. Glob Soc Welf. 2016;3(1):33–43. 10.1007/s40609-015-0039-8

[9] Savarkar T. Psychosocial distress among children living on the Street in Mumbai City, India. J Depress Anxiety. 2018;7(2):308. 10.4172/2167-1044.1000308

[10] Woan J, Lin J, Auerswald C. The health status of street children and youth in low- and middle-income countries: A systematic review of the literature. J Adolesc Health. 2013;53(3):314–321. 10.1016/j.jadohealth.2013.03.013 23706729

[11] Faloore O, Asamu F. Social networks and livelihood of street children in Ibadan, Nigeria. J Int Soc Res. 2010;3(10):487–494.

[12] UNICEF. The state of the world’s children2006: Researching the excluded and invisible children of the world [homepage on the Internet]. 2006 [cited 2019 Mar 21]. Available from: https://www.unicef.org/reports/state-worlds-children-2006

[13] FMOH. State of Nigerian children; children left behind in Nigeria [homepage on the Internet]. 2015 [cited 2021 Nov 15]. Available from: https://nigeria.savethechildren.net/sites/nigeria.savethechildren.net/files/library/State of Nigerian children report.pdf

[14] UN-CRC. Convention on the rights of the child,committee on the rights of the child consideration in Nigeria [homepage on the Internet]. Vol. 40066. 2008 [cited 2019 Mar 21]. Available from: https://www.ecoi.net/en/file/local/1286089/470_1274281179_g1040066.pdf

[15] UNICEF Nigeria-Bar Human Rights Committee. The child rights manual Nigeria [homepage on the Internet]. 2013 [cited 2021 Nov 15]. Available from: http://www.barhumanrights.org.uk/wp-content/uploads/2013/12/The-Child-Rights-Manual_Nigeria-29.10.13.pdf

[16] Dede P, John R. Bronfenbrenner’s ecological systems theory. National-Louis University; c2001 [cited 2018 Dec 10]. Available from: https://dropoutprevention.org/wp-content/uploads/2015/07/paquetteryanwebquest_20091110.pdf.

[17] Härkönen U. The Bronfenbrenner ecological systems theory of human development. Presented at: Scientific articles of V International Conference PERSON.COLOR.NATURE.MUSIC; 2007 Oct 17–21; Latvia: Daugavpils University, Saule; p. 1–19.

[18] Rus AV, Lee WC, Salas BD, et al. Bronfenbrenner’s ecological system theory and the experience of institutionalization of Romanian children. In: Popa C, Lee W, Rus A, editors. New approach behavioral sciences. Târgu Mureș: Editura University Press, 2020; p. 237–251.

[19] Pathak VC. Phenomenological research: A study of lived experiences. Int J Adv Res Innov Ideas Educ. 2017;3(1):1719–1722.

[20] Srivastava A, Thomson S. Framework analysis: A qualitative methodology for applied policy research. JOAAG. 2009;4(2):72–79.

[21] Deitchler M, Ballard T, Swindale A, Coates J. Introducing a simple measure of household hunger for cross-cultural use [homepage on the Internet]. Vol. 2. USAID: Food and Nutrition Technical Assistance; 2011 [cited 2021 Mar 25]. Available from: http://www.fantaproject.org/research/validation-hhs

[22] Owoo NS. Food insecurity and family structure in Nigeria. SSM Popul Health. 2018;4:117–125. 10.1016/j.ssmph.2017.12.004 29349280 PMC5769104

[23] O’Haire H. Living on the streets: The street children of Brazil. Inq J [serial on the Internet]. 2011 [cited 2019 January 15];3(4). Available from: http://www.inquiriesjournal.com/articles/506/living-on-the-streets-the-street-children-of-brazil.

[24] Ayaya SO, Esamai FO. Health problems of street children in Eldoret, Kenya. East Afr Med J. 2001;78(12):624–629. 10.4314/eamj.v78i12.8930 12199442

[25] Alenoma G. Parental perspectives on children streetism in Tamale in Ghana. Res Hum Soc Sci. 2012;2(8):74–82.

[26] Sehra R, Bairwa A, Khatri P, Berwal P, Nagaraj N. A study of nutritional status and common health problems of street children in Bikaner, Rajasthan, India. Int J Community Med Public Health. 2016;3(11):3040–3044. 10.18203/2394-6040.ijcmph20163907

[27] Amury Z, Komba A. Coping strategies used by street children in the event of illness. Research on Poverty Alleviation (REPOA), Dar es Salaam, Tanzania; 2010.

[28] Foundation for Research on Educational Planning and Development (FREPD). A baseline survey of street children in Bangladesh. Dhaka: Dhaka University; 2003.

[29] Ugwuadu ME. Experiences of street children in Iwo Road of Ibadan, Nigeria. Accra: University of Ghana; 2017.

[30] Dybicz P. Interventions for street children. Int Soc Work. 2005;48(6):763–771. 10.1177/0020872805057083

[31] Arthur II. ‘Streetism’ – A socio-cultural and pastoral theological study of a youth problem in Ghana [homepage on the Internet]. Emory University; 2012 [cited 2019 Jan 15]. Available from: https://etd.library.emory.edu/downloads/hd76s0545?locale=zh

[32] Zakaria AFM, Rahman M, Rahman H, Zakaria AFM, Mohammad M-U-H. Street children: Survival on the extreme margins of human life? Int J Hum Soc Sci Educ. 2015;2(9):136–144.

[33] Jamdade VN. A review on the health status of the street children: An exploratory study. Int J Nurs Med Investig. 2016;1(2):128–138.

[34] Pinzon-Rondon AM, Koblinsky SA, Hofferth SL, Pinzón-Florez CE, Briceno L. Work-related injuries among child street-laborers in Latin America: Prevalence and predictors. Rev Panam Salud Publica/Pan Am J Public Health. 2009;26(3):235–243. 10.1590/S1020-49892009000900008 20058834

[35] Jakaza T, Nyoni C, Muzingili T. Emerging dynamics of substance abuse among street children in Zimbabwe. A case of Harare central bussiness district. Afr J Soc Work. 2018;8(2):63–70.

[36] Mhizha S, Tandire J, Muromo T, Matika M. Ecological self-image and behaviours for children living on the streets of Harare. Dev South Afr. 2016;33(1):39–52. 10.1080/0376835X.2015.1113124

[37] Fiasorgbor D. Street children: Our health and coping strategies when we are sick. J Health Med Nurs. 2015;15:45–51.

[38] Van Rooyen L, Hartell CG. Health of the street child: The relation between life-style, immunity and HIV/AIDS – A synergy of research. S Afr J Educ. 2002;22(3):188–192.

[39] Orme J, Seipel MMO. Survival strategies of street children in Ghana: A qualitative study. Int Soc Work. 2007;50(4):489–499. 10.1177/0020872807077909

[40] Fawole O, Ajuwon A, Osungbade K. Violence and HIV/AIDS prevention among female out-of-school youths in southwestern Nigeria: Lessons learnt from interventions targeted at hawkers and apprentices. Afr J Med Med Sci. 2004;33(4):347–353. 15977443

[41] Ennew J. Difficult circumstances: Some reflections on ‘street children’ in Africa. Child Youth Environ. 2003;13(1):1–18.

[42] Myburgh C, Moolla A, Poggenpoel M. The lived experiences of children living on the streets of Hillbrow. Curationis. 2015;38(1):1–8. 10.4102/curationis.v38i1.1274 PMC6091610 26018464

[43] Mwiya LI, Serah B. Mental health problems of street children in residential care in Zambia: Special focus on prediction of psychiatric conditions in street children. J Clin Med Res. 2016;7(1):1–6. 10.5897/JCMR11.025

[44] Wargan K, Dershem L. Don’t call me a street child: Estimation and characteristics of urban street children in Georgia [homepage on the Internet]. 2009 [cited 2019 Jan 15]. Available from: https://resourcecentre.savethechildren.net/pdf/6347.pdf

[45] Aransiola JO. Providing sustainable supports for street children in Nigeria: Stakeholders challenges and the policy options available. Adv Appl Sociol. 2013;3(3):172–177. 10.4236/aasoci.2013.33023

[46] Olaleye Y, Oladeji D. Single parenthood impact on street children in Ibadan Metropolis, Nigeria. Afr Res Rev. 2011;4(2):185–196. 10.4314/afrrev.v4i2.58302

[47] Merril R, Njord L, Njord R, Read C, Pachro J. The effect of family influence on indicators associated with street life among Filipino street children. An Int Interdiscip J Res Policy Care. 2010;5(2):142–150. 10.1080/17450121003615369

[48] Malindi MJ. Exploring the roots of resilience among female street-involved children in South Africa. J Psychol. 2014;5(1):35–45. 10.1080/09764224.2014.11885503

[49] Asante KO. Factors that promote resilience in homeless children and adolescents in Ghana: A qualitative study. Behav Sci. 2019;9(6):64. 10.3390/bs9060064 31216633 PMC6617399

[50] Hills F, Meyer-Weitz A, Asante KO. The lived experiences of street children in Durban, South Africa: Violence, substance use, and resilience. Int J Qual Stud Health Well-being. 2016;11(1):30302. 10.3402/qhw.v11.30302 27291160 PMC4904070

[51] Rus AV, Parris S, Cross D, Purvis K. Romanian institutionalized children’s privation and bronfenbrenner’s ecological system theory, In research, education and development: Symposium proceedings. Cluj-Napoca, Romania: Risoprint Publishing House; 2010.

[52] Nanda S. Working street children’s perceptions of their health, illness and health-seeking behaviour – A qualitative study in New Delhi, India. Amsterdam: Vrije Universiteit; 2008.

[53] Ikechebelu JI, Udigwe GO, Ezechukwu C, Joe-ikechebelu NN. Sexual abuse among juvenile female street hawkers in Anambra State, Nigeria. Afr J Reprod Health. 2008;12(2):111–119. 20695046

